# EYA4 inhibits hepatocellular carcinoma growth and invasion by suppressing NF-κB-dependent RAP1 transactivation

**DOI:** 10.1186/s40880-018-0276-1

**Published:** 2018-04-03

**Authors:** Shi-Jing Mo, Xun Hou, Xiao-Yi Hao, Jian-Peng Cai, Xin Liu, Wei Chen, Dong Chen, Xiao-Yu Yin

**Affiliations:** Department of Pancreatobiliary Surgery, The First Affiliated Hospital, Sun Yat-sen University, No. 58, Zhongshan Er Road, Guangzhou, 510080 Guangdong P. R. China

**Keywords:** Eyes absent homolog 4 (EYA4), RAS-related protein 1 (RAP1), Nuclear factor-κB (NF-κB), Transactivation, Hepatocellular carcinoma

## Abstract

**Background:**

Our previous studies demonstrated that eyes absent homolog 4 (EYA4), a member of the eye development-related EYA family in *Drosophila*, is frequently methylated and silenced in hepatocellular carcinoma (HCC) specimens and associated with shorter survival. The current work aimed to explore the mechanisms through which EYA4 functions as a tumor suppressor in HCC.

**Methods:**

Stable EYA4-expressing plasmid (pEYA4) transfectants of the human HCC cell lines Huh-7 and PLC/PRF/5 (PLC) were established. Xenografts tumors were established via subcutaneous injection of the stable transfectants into BALB/c nude mice. Tissue samples were obtained from 75 pathologically diagnosed HCC patients. Quantitative real-time polymerase chain reaction, Western blotting and immunohistochemistry were performed to determine the expression of EYA4 in cell lines, xenografts and clinical specimens. The cell proliferation, colony formation, invasiveness and tumor formation of stable transfectants were studied. A gene expression microarray was utilized to screen genes regulated by EYA4 expression. The effect of EYA4 on nuclear factor-κB (NF-κB)/RAS-related protein 1 (RAP1) signaling was demonstrated through the co-transfection of pEYA4 and Flag-tagged RAS-related protein 1A gene-expressing plasmid (Flag-RAP1A), functional studies, chromatin immunoprecipitation, immunofluorescence staining and cellular ubiquitination assay.

**Results:**

The restoration of EYA4 expression in HCC cell lines suppressed cell proliferation, inhibited clonogenic outgrowth, reduced cell invasion and restrained xenograft tumor growth, and Flag-RAP1A reversed the suppressive effects of pEYA4 in vitro. Activation of NF-κB with tumor necrosis factor-α (TNF-α) increased the binding of p65 to the RAP1A gene promoter and up-regulated RAP1 protein expression. The inhibition of NF-κB with BAY 11-7085 and p65 siRNA successfully blocked TNF-α-induced RAP1 up-regulation. EYA4 antagonized the TNF-α-induced phosphorylation and ubiquitination of inhibitor of NF-κBα (IκBα) as well as the nuclear translocation and transactivation of p65, resulting in repressed NF-κB activity and RAP1 expression. Blocking the serine/threonine phosphatase activity of EYA4 with calyculin A notably abrogated its suppressive effect on NF-κB activity. In addition, EYA4 expression was inversely correlated with IκBα/RAP1 activity in clinical HCC specimens.

**Conclusion:**

Our findings provide a functional and mechanistic basis for identifying EYA4 as a *bona fide* tumor suppressor that disrupts aberrant activation of the NF-κB/RAP1 signaling pathway and thus orchestrates a physiological impediment to HCC growth and invasion.

## Background

Hepatocellular carcinoma (HCC) is a distinctive digestive malignancy that presents as the sixth most prevalent solid tumor and the second most common cause of cancer-related deaths worldwide [1–3]. An effective therapy for HCC remains a great challenge due to its aggressive behaviors. HCC is usually diagnosed in an advanced stage, at which point potentially curative therapies, such as surgical resection, radiofrequency ablation and liver transplantation, are not always available, and systemic chemotherapy is almost futile [4]. Recent progresses in radiation therapies have provided an alternative treatment approach for patients with unresectable HCC [5]. However, recurrence after treatment remains a major obstacle in improving patient survival. Noticeably, as shown over the past decade, targeted therapy using the tyrosine kinase inhibitor sorafenib represents a notable landmark in the management of HCC because it prolongs the survival of advanced HCC patients and postpones their time-to-progression [6, 7]. Following the triumph of sorafenib, several targeted agents, such as sunitinib [8] and bevacizumab [9], which exhibit antitumor activity by inhibiting the vascular endothelial growth factor (VEGF)/vascular endothelial growth factor receptor (VEGFR) pathway and/or the platelet-derived growth factor (PDGF)/platelet-derived growth factor receptor (PDGFR) pathway, were also found to be effective against HCC. Although progress has been made, the effects of the current targeted therapies remain merely moderate. The search for novel tumor suppressors and therapeutic targets in the complex network of HCC proliferation and progression remains imperative.

The eyes absent homolog (EYA) family, which has four members (EYA1/2/3/4), was initially identified as an eye development-related protein in *Drosophila* characterized by the presence of two critical domains: the tyrosine phosphatase domain in the carboxy terminus and the serine/threonine phosphatase domain in the amino terminus [10, 11]. The dual phosphatase property of the EYA family is not only observed in eye development and organogenesis [12, 13] but also involved in other functions, such as regulating apoptosis/survival decisions [14, 15] and innate immune responses [16]. Moreover, it has also been linked to tumor proliferation, migration and invasion [17]. EYA4 appears to have both tumor-promoting and tumor-suppressing functions in different cellular contexts. For instance, in malignant peripheral nerve sheath tumors, EYA4 prominently sustains cell survival, migration and adhesion [18], and in contrast, emerging evidence implies that EYA4 is most likely a typical tumor suppressor that is epigenetically silenced in various tumors, such as esophageal adenocarcinoma [19], colorectal [20] and pancreatic cancer [21]. Our previous study also revealed that EYA4 expression is repressed in human pancreatic ductal adenocarcinoma and mediates tumor-suppressive effects by abolishing the β-catenin Ser675-dependent transactivation of inhibitor of DNA binding 2 (ID2) [22]. In our previous study, we revealed that the promoter of EYA4 is frequently hypermethylated and persistently down-regulated in human HCC. Moreover, the hypermethylation and silencing of EYA4 are closely correlated with shorter survival [23]. However, the influence of EYA4 on the malignant properties of HCC cells and the underlying mechanisms remain poorly understood.

The aim of this study was to elucidate the potential tumor-suppressive role of EYA4 in HCC and its underlying mechanism. We assessed the effects of EYA4 on cell growth, invasion and tumor formation as well as RAS-related protein 1 (RAP1) expression in HCC. In this study, we also investigated the regulatory effect of EYA4 on the nuclear factor-κB (NF-κB)/RAP1 axis and the underlying mechanism.

## Methods

### HCC specimens, reagents, antibodies, plasmids and primers

The study was approved by the Ethical Committee of the First Affiliated Hospital, Sun Yat-sen University. Seventy-five HCC samples were obtained from the Department of Pancreatobiliary Surgery, The First Affiliated Hospital, Sun Yat-sen University, and with written informed consent obtained from the patients. Recombinant human tumor necrosis factor (TNF-α) was purchased from PeproTech (Rocky Hill, NJ, USA). The NF-κB inhibitor BAY 11-7085 and the serine/threonine phosphatase inhibitor calyculin A (CA) were obtained from Sigma (St. Louis, MO, USA). Rabbit anti-EYA4 (1:200) and mouse anti-RAP1 (1:500) were acquired from Abcam (Cambridge, UK). Rabbit anti-phospho-IκBα-Ser32 (1:2000), anti-phospho-p65-Ser536 (1:1000), anti-phospho-β-catenin-Ser675 (1:1000), anti-p65 (1:1000) and anti-β-catenin (1:1000), mouse anti-IκBα (1:1000) and anti-ubiquitin (1:1000) were obtained from Cell Signaling Technology (Danvers, MA, USA). Rabbit anti-GAPDH (1:3000) and rabbit anti-H2A.X (1:1000) were purchased from Biosynthesis (Beijing, China). Alexa Fluor 594 conjugated anti-rabbit secondary antibody (1:500), horseradish peroxidase (HRP)-linked goat anti-rabbit antibody (1:2000) and horse anti-mouse secondary antibody (1:2000) were procured from Cell Signaling Technology (Danvers, MA, USA). EYA4-expressing plasmid (pEYA4), Flag-tagged human RAP1A-expressing plasmid (Flag-RAP1A) and corresponding empty vectors were purchased from GeneCopoeia (Rockville, MD, USA). EYA4-targeted short hairpin RNA (shEYA4) and scramble short hairpin RNA (Scr) were designed and synthesized by RiboBio (GuangZhou, China). Gene primers for detection of EYA4, RAS-related protein 1A (RAP1A), B cell leukemia/lymphoma 2 (BCL-2), cyclin D1 (CCND1), twist-related protein 1 (TWIST1), X-linked inhibitor of apoptosis (XIAP), glyceraldehyde 3-phosphate dehydrogenase (GAPDH) and RAP1A promoter region were synthesized by Reinjack Bio (Shanghai, China). siRNA targeting p65 (sc-29410) and nontargeting siRNA (sc-37007) were purchased from Santa Cruz (Dallas, TX, USA). The oligonucleotide sequences are listed in Table 1.

**Table 1:** Oligonucleotides used in the study

Target genes	Sequences	Length of product (bp)
shRNA inserts		
EYA4	F: 5′-GATCCCGCACTTAAGTCTTTATCAATTCAAGAGATTGATAAAGACTTAAGTGCTTTTTTCCAAA-3′	64
	R: 5′-AGCTTTTGGAAAAAAGCACTTAAGTCTTTATCAATCTCTTGAATTGATAAAGACTTAAGTGCGG-3′	64
Scramble	F: 5′-GATCCCGTCTCATTATTGCTAAACAGTTGATATCCGCTGTTTAGCAATAATGAGATTTTTTCCAAA-3′	66
	R: 5′-AGCTTTTGGAAAAAATCTCATTATTGCTAAACAGCGGATATCAACTGTTTAGCAATAATGAGACGG-3′	66
qRT-PCR		
EYA4	F: 5′-GAATAACACAGCCGATGG-3′	18
	R: 5′-CCAGGTCACTATCAGGAG-3′	18
RAP1A	F: 5′-CTCCTGAACCAAGGACCACT-3′	20
	R: 5′-TGTGTCTCACTGCACCTTCA-3′	20
BCL-2	F: 5′-GAGAAATCAAACAGAGGCCG-3′	20
	R: 5′-CTGAGTACCTGAACCGGCA-3′	19
CCND1	F: 5′-GGCGGATTGGAAATGAACTT-3′	20
	R: 5′-TCCTCTCCAAAATGCCAGAG-3′	20
TWIST1	F: 5′-TCCATTTTCTCCTTCTCTGGAA-3′	22
	R: 5′-GTCCGCGTCCCACTAGC-3′	17
XIAP	F: 5′-GACCCTCCCCTTGGACC-3′	17
	R: 5′-CTGTTAAAAGTCATCTTCTCTTGAAA-3′	26
GAPDH	F: 5′-AATCCCATCACCATCTTCCA-3′	20
	R: 5′-TGGACTCCACGACGTACTCA-3′	20
ChIP		
RAP1A promoter	F: 5′-CATAAGCATTTACCAGGAAAATGG-3′	24
	R: 5′-GGTTCCTAACAGATGATGGAAA-3′	22

*EYA4* eyes absent homolog 4, *RAP1A* RAS-related protein 1A gene, *BCL-2* B cell leukemia/lymphoma 2, *CCND1* cyclin D1, *TWIST1* twist-related protein 1, *XIAP* X-linked inhibitor of apoptosis, *GAPDH* glyceraldehyde 3-phosphate dehydrogenase, *shRNA* short hairpin RNA, *qRT-PCR* quantitative real-time polymerase chain reaction, *ChIP* chromatin immunoprecipitation

### Cell lines and stable transfection

Huh-7 and PLC/PRF/5 (PLC5) cells were directly obtained from Cell Bank of the Chinese Academy of Sciences (Shanghai, China). The cells were grown in complete DMEM (Invitrogen, Carlsbad, CA, USA) at 37 °C in a humidified atmosphere of 95% air and 5% CO_2_. The cells were obtained within 6 months before use.

The transfection of pEYA4, Flag-RAP1A, shEYA4, Scr, p65 siRNA and nontargeting siRNA was performed using Lipofectamine 2000 (Invitrogen, Carlsbad, CA, USA) according to the manufacturer’s recommended protocol. For the generation of stable transfectants, Huh-7 or PLC5 cells (2 × 10^5^ cells/well) were seeded onto six-well plates. After reaching 80% confluence, the cells were transfected with 4.0 µg of the plasmids using Lipofectamine 2000. After 6 h, the medium was replaced with complete DMEM, and after 48 h, the cells were harvested, selected in complete DMEM containing 800 μg/mL G418 (Merck, Darmstadt, Germany) for 2 weeks and then subjected to limited dilution to isolate and expand the stable pEYA4-transfected HCC cell lines. The stable transfectants were subsequently cultured under selective conditions.

### Cell viability, colony formation and invasiveness

The cell viability was measured using a Cell Counting kit-8 (CCK-8) (Dojindo, Kumamoto, Japan) as described previously [24]. Briefly, the cells were plated into 96-well plates at a density of 3 × 10^3^ cells per well in 100 µL of growth medium and cultured for 24 h. Each day, 10 µL of CCK-8 reagent was added to each well, and the plate was incubated for 2 h according to the manufacturer’s instructions. The absorbance values at 450 nm (A450) were then measured using a Thermo Fisher Scientific microplate reader (Waltham, MA, USA).

For colony formation assays, the cells were plated in six-well plates at 1 × 10^3^ cells per well and allowed to grow in complete DMEM for 10 days. The colonies were then fixed with 4% paraformaldehyde for 5 min, stained with 1% crystal violet for 15 min, rinsed three times with phosphate-buffered saline (PBS), photographed and counted.

The cell invasiveness was evaluated by transwell assays using BioCoat™ Matrigel™ Invasion Chambers (BD Biosciences, San Jose, CA, USA) as previously described [22]. Briefly, the tested cells (1 × 10^5^) were plated on the top side of the polycarbonate transwell filter in the upper chamber and incubated at 37 °C with 5% CO_2_. Twenty-four hours later, the cells inside the upper chamber were removed by cotton swabs, and the invaded cells on the lower surface of the membrane were then fixed with 4% paraformaldehyde, stained with 1% crystal violet, washed with double-distilled dH_2_O and counted. Each independent experiment was performed three times, and the data are presented as the means ± standard deviations (SDs).

### Quantitative real-time polymerase chain reaction (qRT-PCR) and microarray analysis

qRT-PCR experiments were performed as described previously [23]. Briefly, the total RNA from the indicated HCC tissues and cells was extracted using TRIzol^®^ reagent (Invitrogen, Carlsbad, CA, USA) and reverse-transcribed to complementary DNA using the PrimeScript^®^ RT Reagent Kit (TaKaRa, Dalian, China). Each sample was then subjected to real-time PCR using the 7900HT Fast Real-Time PCR system (Applied Biosystems, Waltham, MA, USA) and the SYBR^®^ Premix Ex Taq™ Kit (TaKaRa, Dalian, China) according to the manufacturer’s instructions.

The mRNA was extracted from the stable pEYA4 transfectants and sent for microarray hybridization, data generation, and normalization at Gene Tech (Shanghai, China) using GeneChip^®^ PrimeView™ Human Gene Expression Arrays (Affymetrix, Santa Clara, CA, USA) following standard protocols. The expression data of EYA4-regulated genes in the HCC cell lines were extracted, and a heat map was drawn using HeatMap Builder software (Clifton Watt, Stanford University, CA, USA).

### Immunofluorescence staining for the detection of p65 nuclear translocation

The cells were seeded in 6-well plates and subjected to an immunofluorescence analysis as previously described [16]. In brief, cells plated in six-well plates were fixed with 4% paraformaldehyde in PBS for 10 min at room temperature, permeabilized in 0.5% Triton X-100 for 5 min, washed twice in PBS, and then incubated with 5% bovine serum albumin (BSA; KeyGene Biotech, Nanjing, China) in PBS to block nonspecific binding. After 1 h, the cells were incubated with p65-specific antibody (1:500) overnight at 4 °C and then with Alexa Fluor 594 goat anti-rabbit IgG (1:500) for 1 h at 37 °C. The nuclei were stained with 5 µg/mL DAPI (KeyGene Biotech, Nanjing, China) for 15 min and viewed with an IX71 fluorescence microscope (Olympus, Tokyo, Japan).

### Western blotting of EYA4, RAP1, β-catenin, p65 and IκBα

Western blotting analyses were performed with precast gradient gels (Bio-Rad, Hercules, CA, USA) using standard methods as described previously [15, 17]. Briefly, after isolation, the cytoplasmic and nuclear protein extracts were fractionated through 10% sodium dodecyl sulfate (SDS)–polyacrylamide gel electrophoresis and transferred to Immobilon™ PVDF Transfer Membranes (Millipore, Billerica, MA, USA). After blocking in 5% BSA (KeyGene Biotech, Nanjing, China), the membrane was incubated with specific primary antibodies and then with horseradish peroxidase (HRP)-linked secondary antibody. The bands were visualized using a Western Chemiluminescent HRP Substrate kit (Applygen, Beijing, China). To control sample loading, the blotting membranes were stripped and reprobed with glyceraldehyde 3-phosphate dehydrogenase (GAPDH) or Histone H2A.X antibodies. ImageJ software (http://rsbweb.nih.gov/ij/) was used for densitometric analyses of Western blotting data, and the quantification results were normalized to the loading control.

### Chromatin immunoprecipitation (ChIP) for detecting the binding of p65 to the RAP1A promoter

ChIP assays were performed with EZ-Magna ChIP kit (Millipore, Billerica, MA, USA) according to the manufacturer’s recommended protocol. Briefly, the cells were cross-linked with 1% formaldehyde, washed, lysed, and sonicated. The lysates were immunoprecipitated overnight with gentle rotation at 4 °C using anti-p65 antibody or non-immune rabbit immunoglobulin G as the negative control. Antibody/protein/DNA complexes were coprecipitated with protein-A beads, and the protein-DNA conjugates were eluted with an elution buffer and reversely cross-linked. Standard endpoint PCR was performed using PCR Mastermix (SABioscience, Frederick, MD, USA). The RAP1A promoter domain in immunoprecipitated DNA was amplified and resolved on a 2% agarose/ethidium bromide gel.

### In vivo ubiquitination assay

For in vivo IκBα ubiquitination analysis, 2 × 10^6^ cells were treated with or without 0.1 nmol/L TNF-α for 5 min prior to harvesting. The cells were lysed in radio-immunoprecipitation assay (RIPA) buffer with protease inhibitors and phosphatase inhibitors. IκBα was immunoprecipitated using anti-IκBα agarose at 4 °C for 24 h, and the polyubiquitinated IκBα was then detected using an anti-ubiquitin antibody.

### Immunohistochemistry (IHC)

IHC was performed on formalin-fixed, paraffin-embedded sections as previously described [15]. The percentages of positively stained tumor cells were scored according to the following standards: 0, negative; 1, ≤ 10%; 2, > 10 and < 50%; 3, ≥ 50%. The stained intensity was graded as 0 (negative), 1 (mild), 2 (moderate), or 3 (severe). The staining index (SI) was calculated as the proportion of positive tumor cells × staining intensity. The degree of immunostaining was reviewed and scored independently by two observers who were blinded to the data.

### Tumor growth inhibition assays

BALB/c nude mice (male, 4–6 weeks) purchased from the Guangdong Laboratory Animals Center (GuangZhou, China) were divided into groups of five mice. Huh-7 or PLC5 cells were injected subcutaneously into the right flank as described previously [22]. After tumor formation (diameter > 5 mm), intratumorous transfection with empty vector or pEYA4 was started and repeated each week. The tumors were measured 1 week after the first transfection, and this measurement was repeated weekly. The tumor volume was calculated as 0.52 × (smaller diameter)^2^ × (larger diameter). The mice were sacrificed 4 weeks after the first transfection, and the tumors were then removed and photographed.

For another set of assays, we also inoculated mice directly with stable pEYA4 and vector transfectants. Four weeks after inoculation, the mice were sacrificed, and the tumor weight and EYA4 and RAP1 expression levels were determined.

All animal studies were conducted with the approval of the Sun Yat-sen University Institutional Animal Care and Use Committee and were performed in accordance with established guidelines.

### Statistical analysis

The data are presented as the means ± SDs of at least three independent experiments. Statistical analyses were performed using SPSS 17.0 software (SPSS Inc., Chicago, IL, USA). After a significant result was obtained by ANOVA, statistical comparisons among multiple groups were performed using Bonferroni post hoc *t* tests. Statistical comparisons between two experimental groups were analyzed by unpaired Student’s *t* tests, and a two-tailed *P *< 0.05 was considered to indicate statistical significance.

## Results

### EYA4 overexpression abrogated the malignant properties of HCC cells

**Fig. 1:**
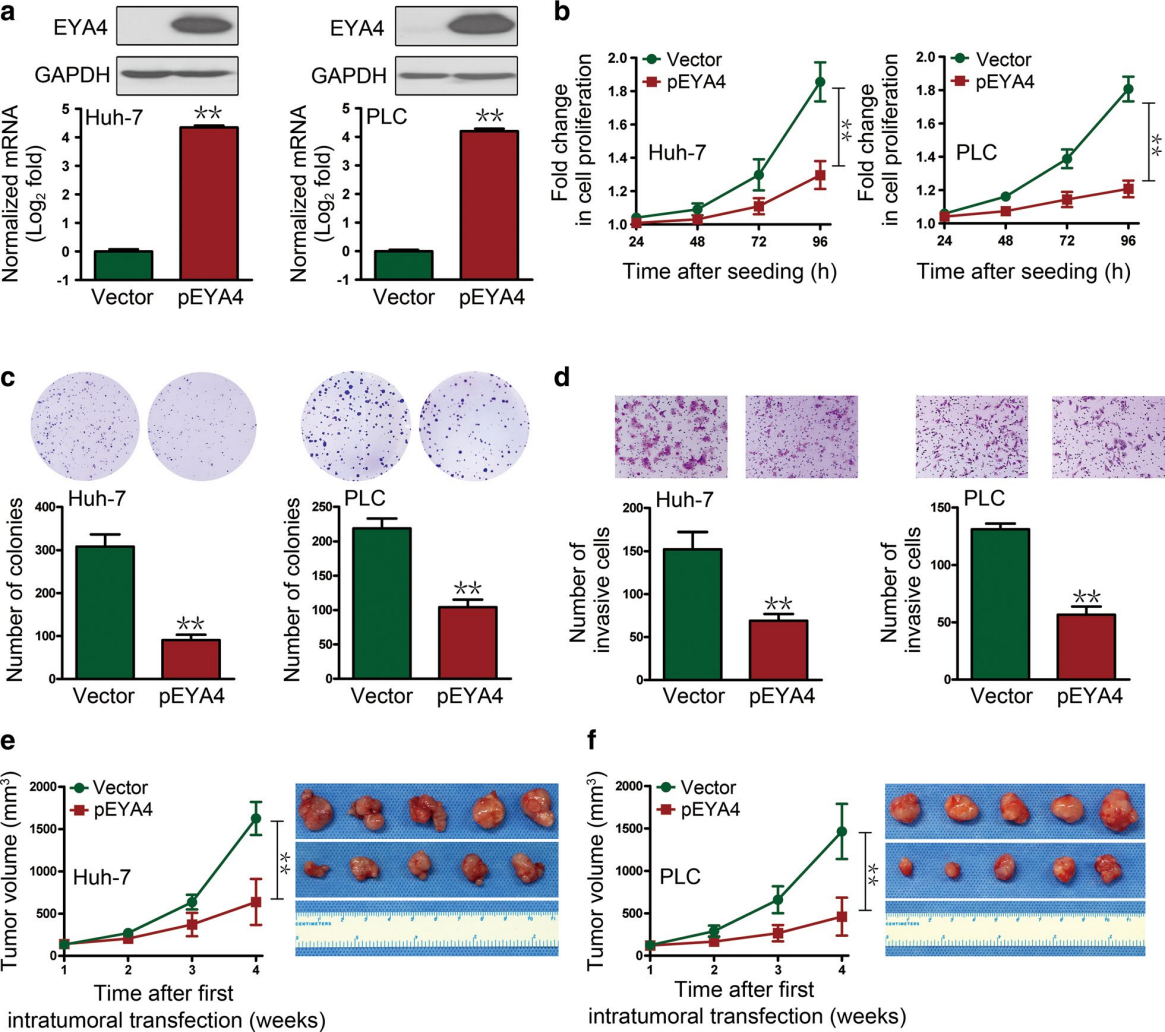
The overexpression of eyes absent homolog 4 (EYA4) antagonized the proliferation, clonogenicity, invasiveness and xenograft tumor growth of human hepatocellular carcinoma (HCC) cells. **a** Quantitative real-time polymerase chain reaction (qRT-PCR) and Western blotting analyses showed elevated EYA4 expression levels in EYA4-expressing plasmid (pEYA) transfectants. **b**–**d** Suppressive effects of EYA4 overexpression on cell proliferation (**b**), colony formation (**c**) and transwell invasion (**d**). **e**, **f** Tumor growth curves (left panel) and images (right panel) of xenografts of Huh-7 (**e**) and PLC5 (**f**) cells after an intratumoral injection of pEYA4. **P < 0.01 between groups. *EYA4* eyes absent homolog 4, *GAPDH* glyceraldehyde 3-phosphate dehydrogenase, *HCC* hepatocellular carcinoma, *pEYA4* EYA4-expressing plasmid, *qRT-PCR* quantitative real-time polymerase chain reaction

To directly elucidate the biological role of EYA4 in HCC cells, we generated stable pEYA4 transfectants of Huh-7 and PLC5 cells. Stable pEYA4 transfectants exhibited markedly increased EYA4 mRNA and protein expression (both *P *< 0.01, Fig. 1a). Notably, significant inhibition of proliferation (both *P *< 0.01, Fig. 1b) and clonogenic outgrowth (both *P *< 0.01, Fig. 1c) was observed in both pEYA4 transfectants compared with the vector transfectants, as demonstrated through CCK8 and colony formation assays, respectively. Consistently, the introduction of pEYA4 led to reduced invasiveness in transwell assays compared with the vector transfectants (both *P *< 0.01, Fig. 1d), indicating that EYA4 exhibits antineoplastic properties in HCC cells. Similarly, intratumoral transfection with pEYA4 greatly impaired subcutaneous tumor growth in nude mice (both *P *< 0.01, Fig. 1e, f). Collectively, these data suggested that EYA4 functions as a *bona fide* tumor suppressor in human HCC cell lines because the overexpression of EYA4 abrogates tumor malignancy.

### EYA4 negatively regulated RAP1

**Fig. 2:**
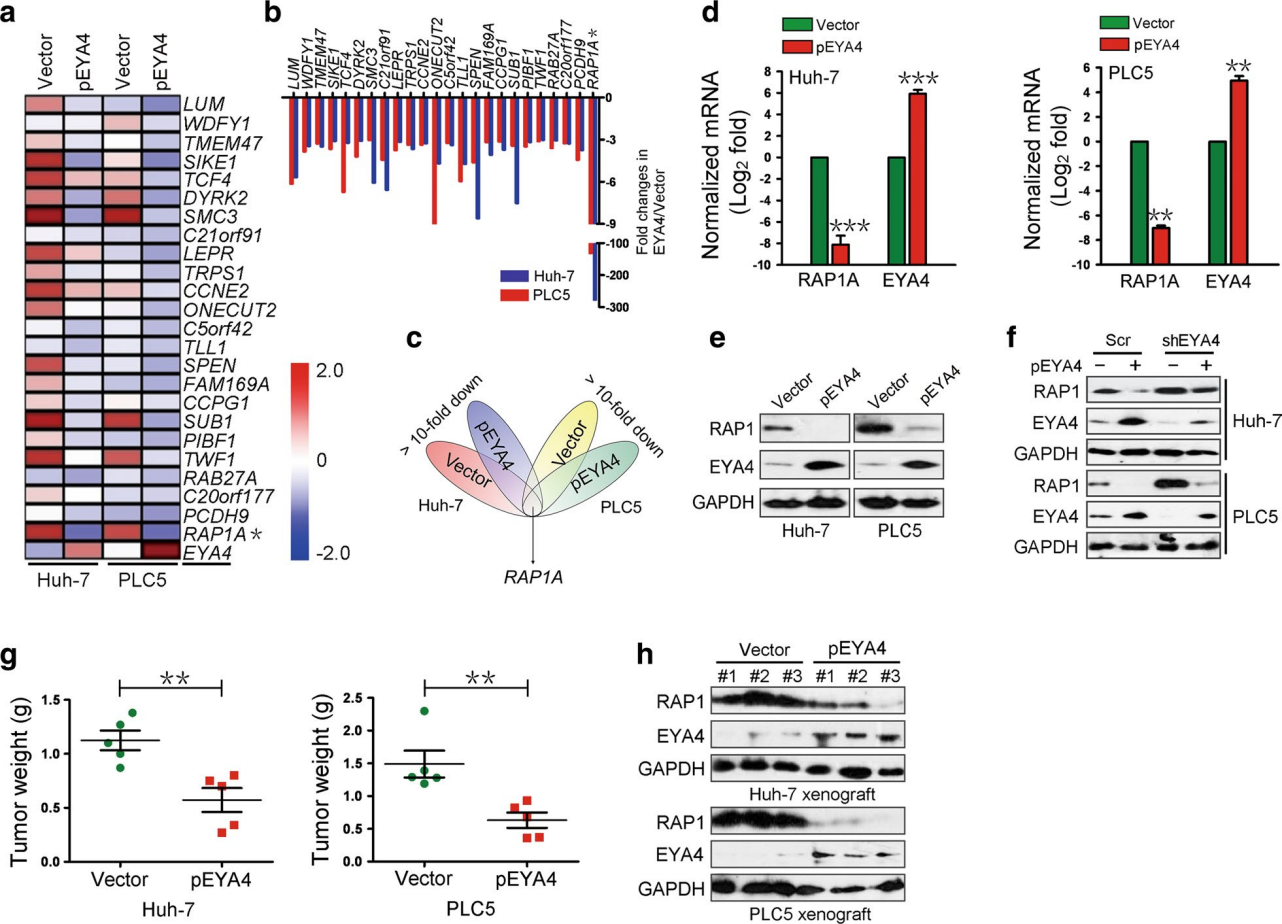
EYA4 negatively regulated RAS-related protein 1 (RAP1) expression in human HCC cells. **a** A heat map of gene expression microarray data shows significantly repressed genes (fold change > 3) in stable pEYA4 transfectants compared with vector transfectants. **b** Comparison of gene expression in the indicated pEYA4 versus vector cells through microarray profiling. **c** Venn diagram for the selection of RAS-related protein 1A gene (RAP1A) using vector- and pEYA4-expressing Huh-7 and PLC5 cells. **d** qRT-PCR analyses of RAP1A and EYA4 mRNA expression in pEYA4 transfectants. **P < 0.01 and ***P < 0.001 versus the vector group. **e** Western blotting analyses of the RAP1 and EYA4 protein expression levels in pEYA4 transfectants. **f** Western blotting analyses of RAP1 protein expression levels in the indicated HCC cells after EYA4 depletion and subsequent pEYA4 transfection. **g** Weight of subcutaneous xenograft tumors formed by pEYA4 and vector transfectants. **P < 0.01 between groups. **h** Western blotting analyses of the EYA4 and RAP1 protein expression levels in the subcutaneous xenograft tumors formed by pEYA4 and vector transfectants. *EYA4* eyes absent homolog 4, *GAPDH* glyceraldehyde 3-phosphate dehydrogenase, *pEYA4* EYA4-expressing plasmid, *RAP1* RAS-related protein 1, *RAP1A* RAS-related protein 1A gene

We then sought to explore the molecular mechanisms through which EYA4 constrains HCC tumorigenesis. The vector and pEYA4 transfectants were subjected to gene expression microarray analyses, and the results showed that 24 genes were notably down-regulated (> threefolds) in both pEYA4 transfectants compared with the corresponding vector transfectants (Fig. 2a, b). One candidate gene, RAP1A, emerged as the most significantly down-regulated gene in EYA4-overexpressing Huh-7 and PLC5 cells (Fig. 2c). Consistently, the qRT-PCR results confirmed that RAP1A mRNA expression was strongly down-regulated in the pEYA4 transfectants compared with the vector transfectants (*P *< 0.001 for Huh-7 cells and *P *< 0.01 for PLC5 cells, Fig. 2d). The RAP1A gene encodes the RAP1 protein. In accordance with the mRNA expression results, the RAP1 protein levels were markedly decreased in both cell lines after pEYA4 transfection (Fig. 2e). In addition, the depletion of EYA4 through shEYA4 transfection potently abrogated the down-regulation of RAP1 protein expression caused by ectopic expression of pEYA4 (Fig. 2f). To investigate whether the tumor-suppressive and RAP1-regulatory effect of EYA4 also occurred in vivo, pEYA4 and vector transfectants from both cell lines were subcutaneously inoculated into the right flank of nude mice to form xenograft tumors. Consistent with the results of the intratumoral transfection study, the tumors of the pEYA4 group weighed significantly less than those of the vector group (both *P *< 0.01, Fig. 2g). Moreover, EYA4-overexpressing xenografts exhibited notably decreased RAP1 protein levels compared with the control tumors (Fig. 2h). Thus, these data validated the finding that EYA4 represses RAP1A mRNA and RAP1 protein expression in vitro and in vivo.

### EYA4 mediated tumor suppression by repressing RAP1

**Fig. 3:**
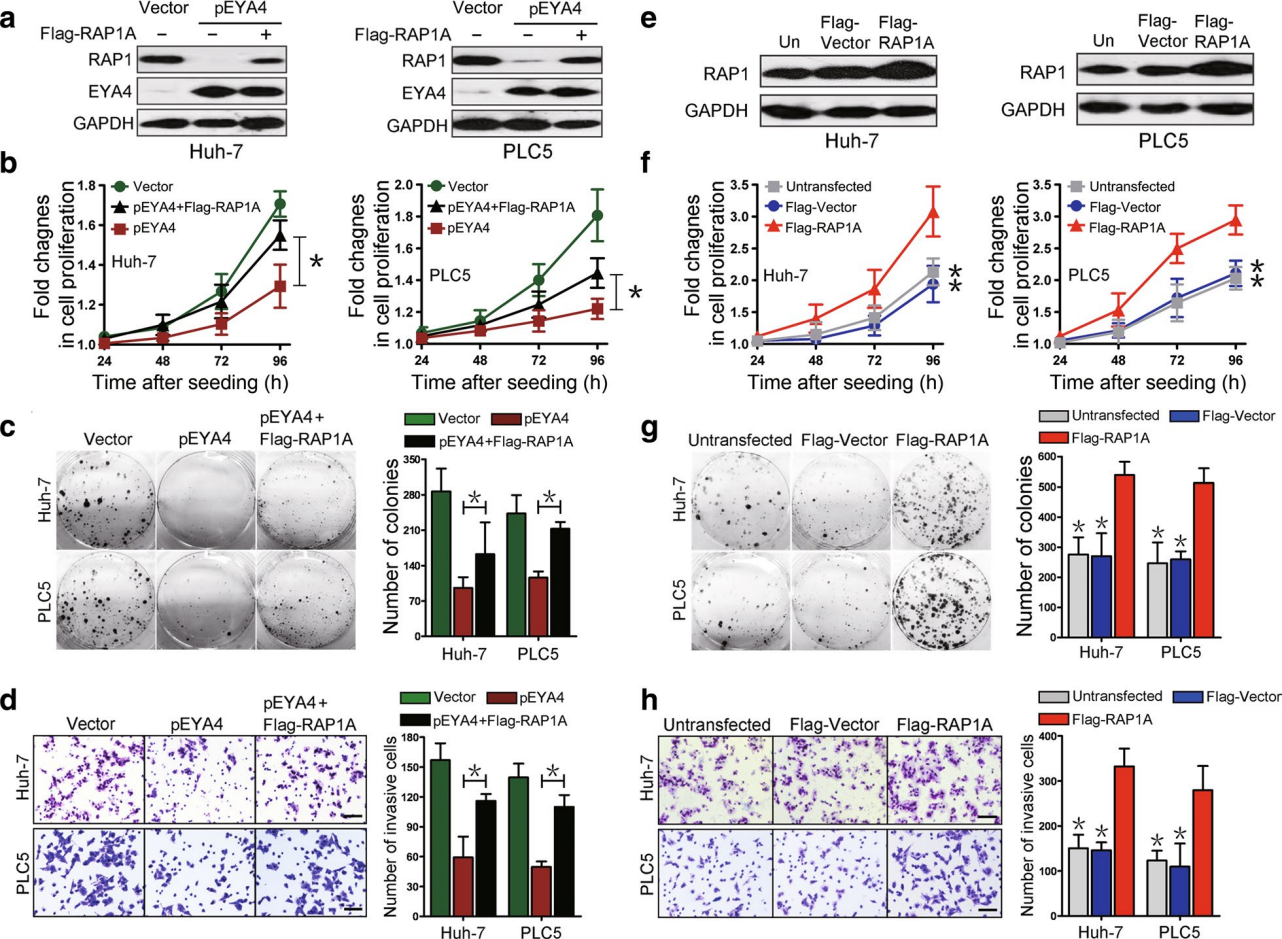
EYA4 inhibited proliferation, clonogenicity and invasiveness of HCC cells by the suppression of RAP1. **a** Western blotting analyses showing the RAP1 protein expression levels in pEYA4 transfectants (left: Huh-7, right: PLC5) after transfection with or without Flag-tagged human RAP1A expressing plasmid (Flag-RAP1A). **b**–**d** Effects of RAP1 expression on cell proliferation (**b**), clonogenicity (**c**) and cell invasiveness (**d**) of EYA4-overexpressing HCC cells. pEYA4 transfectants were transfected with or without Flag-RAP1A, and the resulting cell proliferation, clonogenic outgrowth and cell invasiveness were determined by CCK-8 assays, colony formation assays and transwell invasion assays, respectively. *P < 0.05 between groups. **e** Western blotting analyses of the RAP1 expression levels in Huh-7 (left panel) and PLC5 (right panel) with or without Flag-RAP1A transfection. **f**–**h** Effects of RAP1 expression on cell proliferation (**f**), clonogenicity (**g**) and cell invasiveness (**h**) of HCC cells. The effects were analyzed by CCK-8 assays, colony formation assays and transwell invasion assays, respectively. *P < 0.05 versus the Flag-RAP1A group. *EYA4* eyes absent homolog 4, *Flag-RAP1A* Flag-tagged human RAP1A expressing plasmid, *Flag-Vector* Flag-tagged vector plasmid, *GAPDH* glyceraldehyde 3-phosphate dehydrogenase, *pEYA4* EYA4-expressing plasmid, *RAP1* RAS-related protein 1, *RAP1A* RAS-related protein 1A gene, *Un* untransfected control

To determine whether RAP1 repression is the critical molecular mechanism mediating the tumor-suppressive effects of EYA4 in HCC cells, we co-transfected HCC cells with pEYA4 and Flag-RAP1A. Enforced expression of EYA4 notably repressed RAP1 protein expression, and this effect was clearly reversed by the introduction of ectopic Flag-RAP1A expression in both cell lines (Fig. 3a). Cell proliferation, as measured by growth curves, and clonogenicity, as measured by colony formation assays, were reduced by pEYA4 transfection, but this inhibition was significantly rescued by concurrent transfection with Flag-RAP1A (*P *< 0.05, Fig. 3b, c). The transfection of Huh-7 and PLC5 cells with pEYA4 markedly impaired cell invasiveness in transwell assays, but this effect was also strikingly reversed by concomitant transfection with Flag-RAP1A (*P *< 0.05, Fig. 3d). These data suggested that the antineoplastic effects of EYA4 in HCC cellular phenotypes were mediated by the repression of RAP1.

To test whether RAP1 is sufficient to promote HCC cell growth and invasiveness, Huh-7 and PLC5 cell lines were transfected with Flag-RAP1A, and the results revealed that RAP1 protein expression was up-regulated after Flag-RAP1A transfection in both cell lines (Fig. 3e). CCK8 assays showed a marked increase in cell proliferation in the Flag-RAP1A-transfected cells compared with the cells transfected with empty vectors (*P *< 0.05, Fig. 3f), supporting the notion that RAP1 could contribute to the proliferation of HCC cells. Notably, the cells transfected with Flag-RAP1A also showed significantly increased surviving colony numbers in comparison with the vector controls (*P *< 0.05, Fig. 3g). Consistently, the expression of RAP1 remarkably enhanced the cell invasive ability of both Flag-RAP1A transfectants (*P *< 0.05, Fig. 3h), indicating that RAP1 protein plays an important role in the tumorigenicity of HCC cells. Together, these findings revealed that EYA4 counteracted the malignant properties of HCC cells by repressing RAP1 protein, which was essential for triggering various tumorigenic phenotypes.

### RAP1 was transcriptionally regulated by NF-κB in an EYA4-dependent manner

**Fig. 4:**
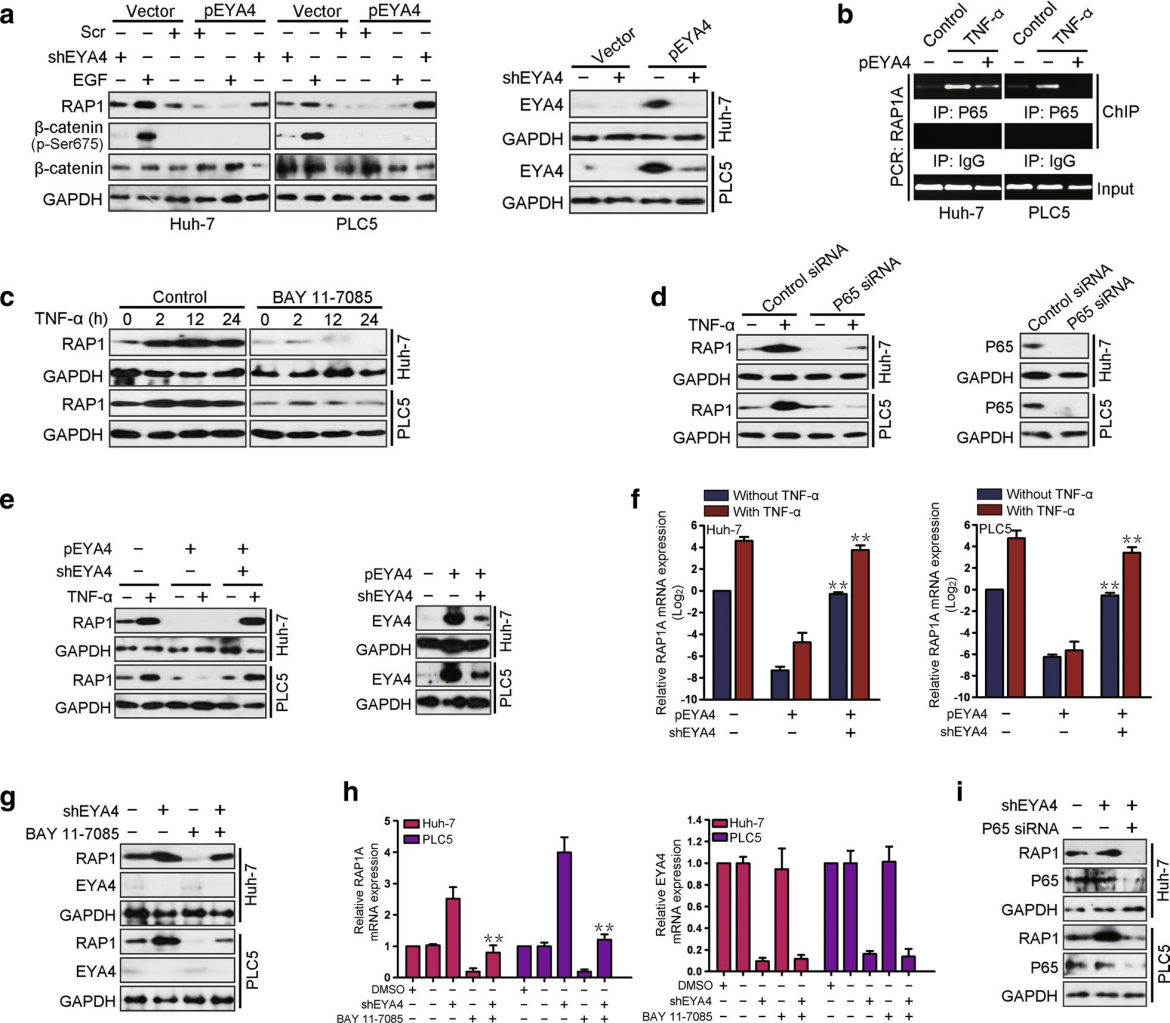
Nuclear factor-κB (NF-κB) regulated RAP1 in an EYA4-dependent manner. **a** Western blotting analyses comparing the levels of RAP1 protein expression in Huh-7 and PLC5 cells after activation of β-catenin with epidermal growth factor (EGF, 100 ng/mL) or silencing of EYA4 with EYA4-targeted short hairpin RNA (shEYA4) in the presence or absence of pEYA4 transfection. **b** Chromatin immunoprecipitation (ChIP) analyses were performed to test the interactions between p65 and the RAP1A promoter in Huh-7 and PLC5 cells with or without pEYA4 transfection upon tumor necrosis factor-α (TNF-α, 10 ng/mL) stimulation. **c** Western blotting analyses comparing the levels of RAP1 protein expression in Huh-7 and PLC5 cells with TNF-α stimulation for the indicated times in the presence or absence of the NF-κB inhibitor BAY 11-7085 (5 µmol/L). **d** Western blotting analyses comparing the levels of RAP1 protein expression in Huh-7 and PLC5 cells upon TNF-α stimulation with or without p65-targeting small interfering RNA (p65 siRNA) transient transfection. **e**, **f** The expression of RAP1 protein (**e**) and RAP1A mRNA (**f**) was analyzed in Huh-7 and PLC5 cells transfected with pEYA4 alone or with pEYA4 plus shEYA4 in the presence or absence of TNF-α stimulation. **P < 0.01 vs. the pEYA4 group. **g**, **h** RAP1 and EYA4 protein expression (**g**) and RAP1A and EYA4 mRNA expression (**h**) in Huh-7 and PLC5 cells in which EYA4 was depleted by shEYA4 in the presence or absence of the NF-κB inhibitor BAY 11-7085. **P < 0.01 vs. shEYA4 group. **i** Western blotting analyses detecting the RAP1 protein abundance in Huh-7 and PLC5 cells in which EYA4 was silenced by shEYA4 transfection in the presence or absence of p65 siRNA transient transfection. The qRT-PCR experiments were performed five times with technical duplicates, and the real-time values were normalized to glyceraldehyde 3-phosphate dehydrogenase (GAPDH). *ChIP* chromatin immunoprecipitation, *DMSO* dimethyl sulfoxide, *EGF* epidermal growth factor, *EYA4* eyes absent homolog 4, *Flag-RAP1A* Flag-tagged human RAP1A expressing plasmid, *Flag-Vector* Flag-tagged vector plasmid, *GAPDH* glyceraldehyde 3-phosphate dehydrogenase, *IgG* immunoglobulin G, *IP* immunoprecipitation, *NF-κB* nuclear factor-κB, *P65* NF-κB p65 subunit, *PCR* polymerase chain reaction, *pEYA4* EYA4-expressing plasmid, *RAP1* RAS-related protein 1, *Scr* scramble short hairpin RNA, *shEYA4* EYA4-targeted short hairpin RNA, *siRNA* small interfering RNA, *TNF-α* tumor necrosis factor-α

We subsequently attempted to examine the mechanisms through which RAP1 expression is repressed by EYA4. Because our previous studies demonstrated that EYA4 dephosphorylates β-catenin Ser675 in human pancreatic ductal adenocarcinoma [22], we explored whether β-catenin Ser675 dephosphorylation is instrumental for RAP1 repression by EYA4 in HCC. As shown in Fig. 4a, RAP1 protein expression was significantly suppressed in the pEYA4 transfectants compared with the vector transfectants. Both EGF-induced β-catenin Ser675 phosphorylation and shEYA4 up-regulated the levels of RAP1 protein expression in the vector-transfected cells. However, in contrast to shEYA4, EGF induction did not restore the reduction in RAP1 protein expression observed in the pEYA4 transfectants, indicating that the RAP1 repression caused by pEYA4 transfection was not due to EYA4-mediated β-catenin Ser675 dephosphorylation.

An analysis of the RAP1A gene promoter revealed a single putative NF-κB-binding sequence, -3678GGGAATTTCC-3669, which was similar to the consensus NF-κB-binding sequence GGGRNNYYCC (R, purine; N, any base; and Y, pyrimidine) [25]. To determine whether RAP1A is a direct downstream target of NF-κB transactivation, we performed ChIP analyses with anti-p65 antibodies. The results showed that the stimulation of TNF-α, a key pro-inflammatory cytokine that is well known to be responsible for elevating NF-κB activity, increased the binding of p65 to the promoter region of RAP1A, and this effect was blocked by the ectopic expression of pEYA4 (Fig. 4b). To evaluate whether NF-κB activation plays a pivotal role in the regulation of RAP1 expression, we detected RAP1 protein expression in Huh-7 and PLC5 cells treated with TNF-α, and as shown in Fig. 4c, TNF-α administration up-regulated RAP1 protein expression in both cell lines after 2 h. In contrast, the treatment of Huh-7 and PLC5 cells with the NF-κB inhibitor BAY 11-7085 successfully blocked the TNF-α-induced up-regulation of RAP1 protein, suggesting that RAP1 was up-regulated by NF-κB activation. This finding was further validated by the observation that p65 depletion by p65 siRNA in both Huh-7 and PLC5 cells strongly abolished the TNF-α-induced RAP1 up-regulation (Fig. 4d), indicating that NF-κB activation was essential for this up-regulation. To test whether EYA4 negatively regulated the ability of NF-κB to transactivate RAP1, we assessed the expression of RAP1A mRNA and RAP1 protein (RAP1A/RAP1) in TNF-α-stimulated cells transfected with pEYA4 alone or with both pEYA4 and shEYA4. The ectopic expression of pEYA4 completely blocked TNF-α-induced RAP1A/RAP1 up-regulation, whereas the depletion of EYA4 by shEYA4 in pEYA4-expressing cells restored the ability of TNF-α to enhance RAP1A/RAP1 expression (Fig. 4e, f). To directly determine whether NF-κB activation is involved in EYA4-mediated RAP1 repression, we treated shEYA4-expressing Huh-7 and PLC5 cells with BAY 11-7085. The depletion of EYA4 with shEYA4 significantly up-regulated the levels of RAP1A/RAP1 expression in HCC cells, but RAP1A/RAP1 expression and the shEYA4-mediated up-regulation of RAP1A/RAP1 were significantly hampered in cells treated with BAY 11-7085 (Fig. 4g, h). In accordance with this finding, the knockdown of p65 by p65 siRNA effectively attenuated the EYA4 depletion-mediated up-regulation of RAP1 protein expression in HCC cells (Fig. 4i), implying a critical role for NF-κB in the EYA4-dependent repression of RAP1 transactivation.

### The serine/threonine phosphatase activity of EYA4 inhibited NF-κB activation

**Fig. 5:**
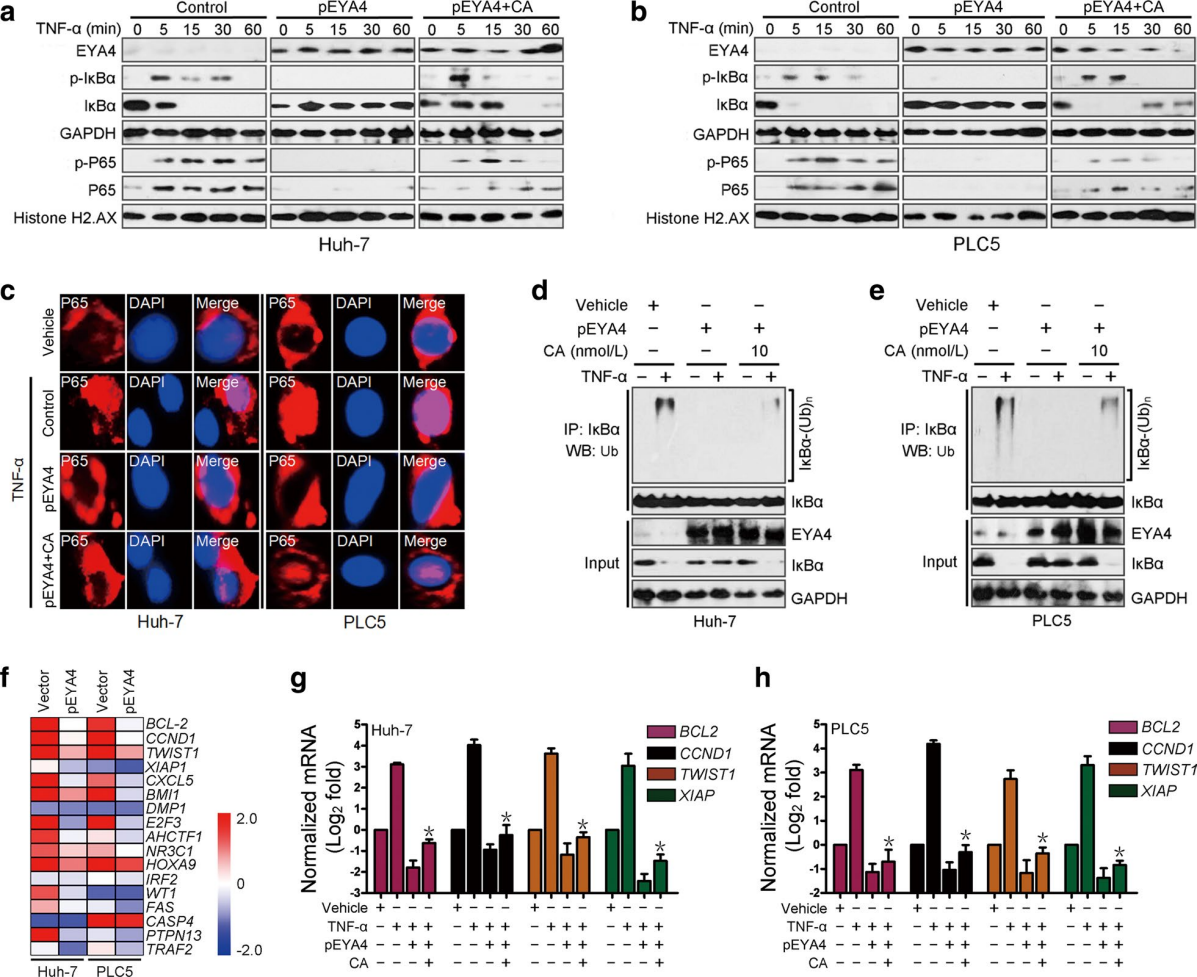
The serine/threonine-phosphatase activity of EYA4 inactivated NF-κB signaling. **a**, **b** Western blotting analyses examining the levels of IκBα phosphorylation and p65 nuclear translocation in Huh-7 (**a**) and PLC5 (**b**) cells transduced with pEYA4 in the presence or absence of the serine/threonine phosphatase inhibitor calyculin A (CA; 10 nmol/L) upon TNF-α (10 ng/mL) stimulation. Histone H2.AX served as the loading control for the nuclear fractions. **c** Representative immunofluorescence staining images comparing the nuclear translocation of p65 in Huh-7 and PLC5 cells transfected with pEYA4 in the presence or absence of CA treatment upon TNF-α stimulation. Scale bar = 20 µm. **d**, **e** In vivo ubiquitination assays comparing the polyubiquitination levels of IκBα in Huh-7 (**d**) and PLC5 (**e**) cells transfected with pEYA4 in the presence or absence of CA treatment upon TNF-α stimulation. **f** EYA4 opposed the transcriptional programs of NF-κB signaling in HCC cells. Heat map of gene expression microarray data showing the expression of NF-κB signaling-dependent genes in the indicated HCC cells. **g**, **h** qRT-PCR analyses of the NF-κB downstream target genes B cell leukemia/lymphoma 2 (BCL-2), cyclin D1 (CCND1), twist-related protein 1 (TWIST1), and X-linked inhibitor of apoptosis (XIAP) in Huh-7 (**g**) and PLC5 (**h**) cells transfected with pEYA4 in the presence or absence of CA upon TNF-α stimulation. The experiments were performed five times, each qRT-PCR assay was performed in technical duplicates, and the real-time values were normalized to GAPDH. *P < 0.05 versus the TNF-α plus pEYA4 groups. *BCL-2* B cell leukemia/lymphoma 2, *CA* calyculin A, *CCND1* cyclin D1, *DAPI* 4′,6-diamidino-2-phenylindole, *EYA4* eyes absent homolog 4, *GAPDH* glyceraldehyde 3-phosphate dehydrogenase, *IκBα* nuclear factor of kappa light polypeptide gene enhancer in B-cells inhibitor, alpha, *IP* immunoprecipitation, *NF-κB* nuclear factor-κB, *P65* NF-κB p65 subunit, *p-IκBα* phosphorylated IκBα, *p-P65* phosphorylated P65, *PCR* polymerase chain reaction, *pEYA4* EYA4-expressing plasmid, *RAP1* RAS-related protein 1, *shEYA4* EYA4-targeted short hairpin RNA, *siRNA* small interfering RNA, *TNF-α* tumor necrosis factor-α, *TWIST1* twist-related protein 1, *Ub* ubiquitination, *(Ub)*_*n*_ polyubiquitination, *WB* Western blotting, *XIAP* X-linked inhibitor of apoptosis

To determine whether EYA4 is capable of inactivating NF-κB signaling, we stimulated pEYA4 and vector transfectants with TNF-α and assessed the influence of EYA4 on TNF-α-induced NF-κB activation. As shown in the left columns of Fig. 5a, b, the levels of phosphorylated IκBα in the cytosolic fractions and the levels of phosphorylated p65 and total p65 in the nuclear fractions were markedly increased 5 min after TNF-α stimulation in the vector transfectants, and TNF-α administration progressively decreased the levels of total IκBα in the cytoplasm, which is consistent with the notion that IκBα phosphorylation activates ubiquitination and degradation [26, 27]. In contrast, the reconstituted expression of pEYA4 in Huh-7 and PLC5 cells almost completely abolished IκBα phosphorylation, abrogated TNF-α-induced IκBα degradation and blocked TNF-α-triggered p65 nuclear translocation (middle columns, Fig. 5a, b). In accordance with the notion that the serine/threonine phosphatase activity of EYA4 is critical for its biological functions [16], pretreatment with calyculin A (CA), a serine/threonine phosphatase inhibitor, largely mitigated the EYA4-abolished phosphorylation and degradation of IκBα and p65 nuclear translocation upon TNF-α stimulation (right columns, Fig. 5a, b). In addition, immunofluorescence staining showed that the TNF-α-induced nuclear translocation of p65 was lost in pEYA4-expressing cells but partially retained in the cells cotreated with CA (Fig. 5c). These results suggest that serine/threonine phosphatase activity was, at least in part, required for the EYA4-mediated inactivation of NF-κB. Concordantly, the introduction of pEYA4 expression greatly reduced the TNF-α-induced polyubiquitination levels of IκBα in Huh-7 and PLC5 cells, but this reduction was successfully reversed by CA (Fig. 5d, e), indicating that the serine/threonine phosphatase activity of EYA4 attenuated Ub conjugation of IκBα. Intriguingly, a microarray analysis of EYA4-dependent gene expression changes in the two HCC cells revealed repression of documented downstream genes of the NF-κB pathway [26, 28] (Fig. 5f). The qRT-PCR results confirmed that enforced EYA4 expression consistently down-regulated four NF-κB downstream target genes, namely, B cell leukemia/lymphoma 2 (BCL-2), cyclin D1 (CCND1), twist-related protein 1 (TWIST1), and X-linked inhibitor of apoptosis (XIAP), in TNF-α-stimulated conditions, whereas treatment with CA effectively restored the expression of these genes (Fig. 5g, h). Collectively, these data indicated that the serine/threonine phosphatase activity of EYA4 dephosphorylated IκBα, counteracted IκBα ubiquitination and degradation, blocked p65 nuclear translocation and thus inactivated NF-κB signaling, which have documented roles in the tumorigenesis of HCC cells.

### EYA4 was inversely associated with the hyperactivated NF-κB/RAP1 signaling axis in clinical HCC samples

**Fig. 6:**
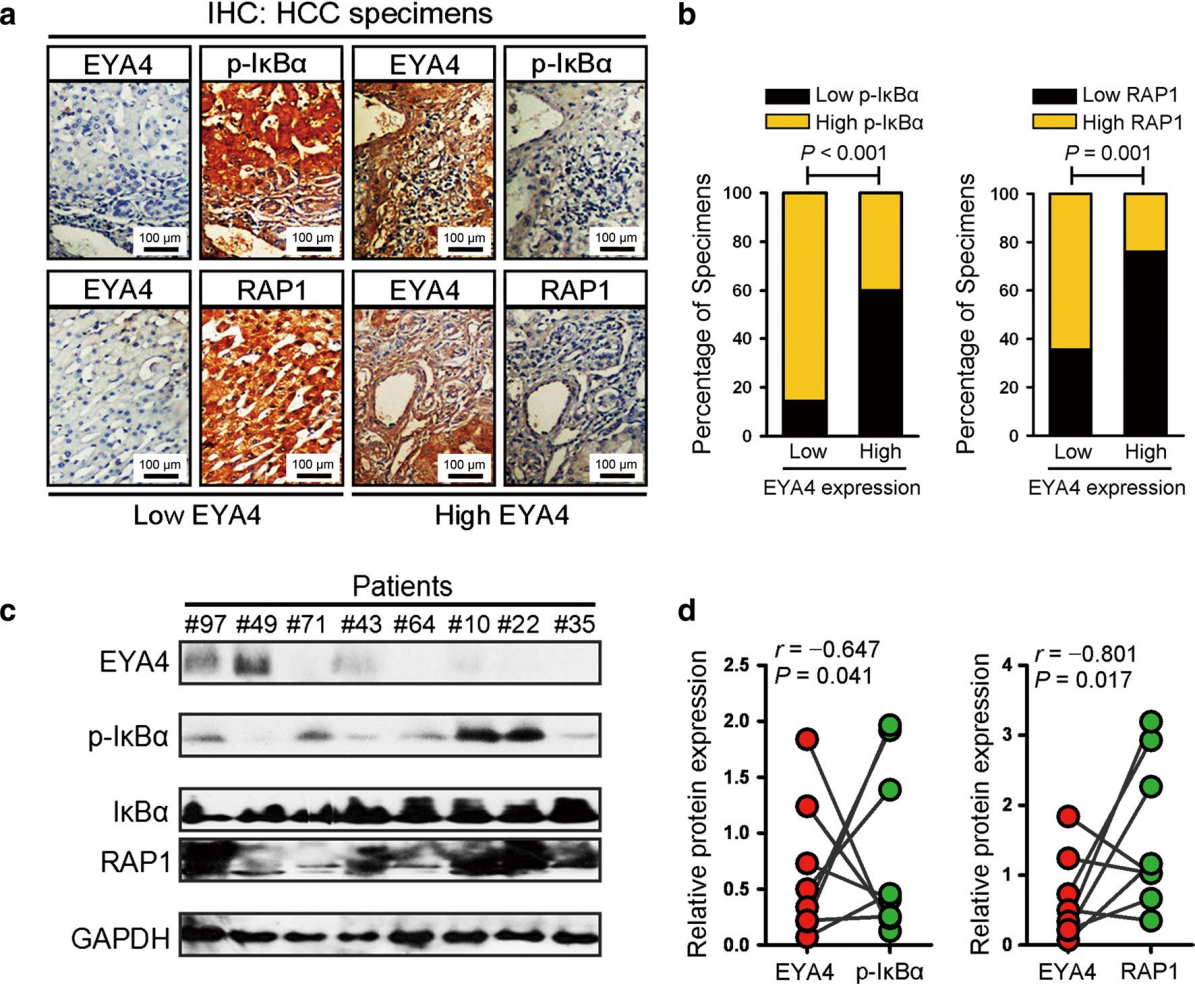
Clinical relevance of EYA4 expression for the IκBα/RAP1 axis in human HCC tissues. **a**, **b** Representative immunohistochemistry images (**a**) and bar graphs (**b**) showing the relevance of EYA4 expression on the phospho-IκBα-Ser32 and RAP1 expression levels in 67 clinical HCC specimens. **c**, **d** Western-blotting analyses (**c**) and correlation analyses (**d**) were used to examine whether the EYA4 protein levels are associated with the levels of phospho-IκBα-Ser32 and RAP1 in eight HCC samples. *EYA4* eyes absent homolog 4, *IκBα* nuclear factor of kappa light polypeptide gene enhancer in B-cells inhibitor, alpha, *GAPDH* glyceraldehyde 3-phosphate dehydrogenase, *p-IκBα* phosphorylated IκBα, *RAP1* RAS-related protein 1

We then sought to assess the association between EYA4 and NF-κB signaling and subsequent RAP1 transactivation in clinical human HCC specimens. To this end, tumor specimens from 67 HCC patients were collected in our current study for IHC analyses using EYA4, p-Ser32-IκB and RAP1 antibodies. The results revealed that 37.3% (25 cases) were positive and 62.7% (42 cases) were negative for EYA4, indicating that EYA4 is mostly silenced in most patients with HCC. By analyzing these HCC tissue specimens, we found that, as shown in Fig. 6a, b, 85.7% and 64.3% of the samples with low EYA4 expression exhibited high levels of p-Ser32-IκB and RAP1, whereas 60.0% and 76.0% of the samples with high EYA4 expression showed low expression of p-Ser32-IκB and RAP1, respectively. These data indicate that EYA4 expression is negatively associated with the levels of p-Ser32-IκB and RAP1 in human HCC specimens. Moreover, EYA4 protein expression in eight freshly collected clinical HCC samples was inversely correlated with the p-Ser32-IκB and RAP1 protein expression levels (Fig. 6c, d). Taken together, our data indicate that EYA4 deficiency in HCC pathogenesis was associated with the hyperactivated NF-κB signaling pathway and up-regulated RAP1 expression, which further contributed to the aggressiveness of malignant liver tumors.

## Discussion

In the present study, we found that the ectopic expression of pEYA4 inhibited HCC cell proliferation, colony formation, invasion and tumor formation by repressing the expression of the RPA1A gene and its protein product RAP1. The investigation showed that the RAP1A gene was transactivated by NF-κB, and this effect was negatively regulated by EYA4. The serine/threonine-specific phosphatase activity of EYA4 might play a critical role in this suppression.

In a previous research, we demonstrated that EYA4 expression is down-regulated in human HCC tissue and that its suppression is an independent prognostic factor of poor survival, which suggests EYA4 might be a potential tumor suppressor gene in HCC [23]. Therefore, stable pEYA4 transfectants were established in the present study to investigate the effect of EYA4 on HCC. Notable suppression of cell proliferation, colony formation and invasiveness in vitro and retarded xenograft tumor growth in vivo were observed in the pEYA4 transfectants. To reveal the mechanisms underlying the abovementioned suppression, the pEYA4 and vector transfectants were analyzed through a gene expression microarray, and the results showed that RAP1A gene expression was notably suppressed by EYA4 overexpression. Moreover, EYA4 expression was negatively correlated with RAP1 expression in surgically resected HCC tissues. A literature review indicated that RAP1 is involved in the malignant progression of tumors from various origins. Chen et al. [29] demonstrated that RAP1 is constitutively activated in oral carcinoma and that its sustained activation is correlated with shorter overall survival of patients. Bailey et al. [30] found that increased RAP1 activity is tightly associated with the metastatic ability of prostate cancer cell lines. Notably, in glioblastoma cell lines, Sayyah et al. [31] observed that RAP1 serves as a vital tumor mediator that is up-regulated in response to thrombin administration. Consistently, we found that RAP1 markedly promoted HCC cells to proliferate, form colonies and invade, thus reinforcing the notion that RAP1 is a powerful proto-oncogene in human HCC. Together with the evidence that EYA4 negatively regulated the levels of RAP1 mRNA and protein expression, we further dissected the role of RAP1 in EYA4-mediated tumor suppression by gain-of-function with re-expression of RAP1. Of note, the impaired malignant phenotypes observed in EYA4-expressing HCC cells were greatly restored by reconstituted expression of wild-type RAP1. Adding to our extensive analysis of the relationship between EYA4 and RAP1 in malignant phenotypes, our results suggest that EYA4 implements the broad antineoplastic effects by repressing RAP1 transactivation.

Niola et al. [32] found that RAP1 function is regulated by ID proteins in mesenchymal high-grade glioma, although the precise mechanism remained unclear. In another study, we revealed that the tumor-suppressive role of EYA4 in pancreatic tumor growth relies on repression of the transactivation of ID2 via β-catenin Ser675 phosphorylation [22]. However, in the present study, although EGF stimulation induced β-catenin Ser675 phosphorylation and increased RAP1 expression in vector cells, it failed to rescue RAP1 expression in EYA4-overexpressing cells, indicating that EYA4 might not rely on β-catenin Ser675 phosphorylation to repress RAP1 in HCC.

NF-κB is an inducible transcriptional factor that has been intensively studied over the past decades. In the classical pathway, NF-κB is released upon IκB phosphorylation and degradation and then translocates to the nucleus to bind to specific promoter sequences and activate transcription [27]. Various studies have clearly linked NF-κB to inflammation and various tumor types, including HCC [33–35]. We noted an NF-κB-binding sequence in the promoter zone of the RAP1A gene and thus tested whether EYA4 repressed RAP1 through the NF-κB pathway. Interestingly, our findings revealed that EYA4 overexpression markedly abrogated IκBα phosphorylation, IκBα polyubiquitination, subsequent p65 nuclear translocation, and the binding of p65 to RAP1A promoter induced by TNF-α. The mechanism through which EYA4 blocks these effects has not been previously documented. Because EYA4 exhibits serine/threonine phosphatase activity [21], we hypothesized that EYA4 dephosphorylates IκBα to suppress NF-κB release and activation. Our study demonstrated that blockage of the serine/threonine phosphatase activity of EYA4 with CA notably reversed the suppression of NF-κB, including multiple downstream target genes, which suggests that EYA4-mediated deactivation of NF-κB might be, at least partly, dependent on its serine/threonine phosphatase activity. Intriguingly, the expression of EYA4 was inversely correlated with the p-Ser32-IκB and RAP1 levels in human HCC specimens, in agreement with our postulation that EYA4 deficiency is a major cause of the hyperactivated state of the NF-κB/RAP1 signaling axis in HCC.

The present study has two limitations. First, we used the serine/threonine phosphatase inhibitor CA to demonstrate that the serine/threonine phosphatase activity of EYA4 was critical for the inhibition of NF-κB signaling in HCC. A serine/threonine phosphatase-dead mutant of EYA4 might provide more convincing evidence. Second, further investigations are needed to elucidate the direct interactions between EYA4 and NF-κB.

## Conclusions

Our current study unearthed EYA4 as a novel tumor suppressor that was suppressed in HCC cell lines. EYA4 inhibited HCC cell growth, invasion and tumor formation by repression of RAP1, highlighting the important role of RAP1 in EYA4-mediated tumor suppression in HCC development. The evidence of a mechanistic interplay between EYA4 deficiency and NF-κB transactivation-dependent RAP1 up-regulation provided a novel insight for further understanding HCC aggressiveness and might offer a molecular rationale for treating EYA4-deficient and/or NF-κB-hyperactive tumors by interfering with this EYA4-dependent signaling transmission at multiple aspects.
